# The mortality of *Bombyx mori* larvae challenged by BmNPV is reduced when supplemented with *Lactobacillus acidophilus* bacteria

**DOI:** 10.1186/s13104-024-07019-9

**Published:** 2024-12-17

**Authors:** Siripuk Suraporn, Vallaya Suthikhum, Olle Terenius

**Affiliations:** 1 Department of Biology, Faculty of Science, Mahasarakham University, Kantarawichai District, Mahasarakham, 44150 Thailand; 2 Center of Excellence for Mulberry and Silk, Mahasarakham University, Kantharawichai District, Mahasarakham, 44150 Thailand; 3 Department of Chemistry, Faculty of Science, Mahasarakham University, Kantarawichai District, Mahasarakham, 44150 Thailand; 4 Department of Cell and Molecular Biology, Uppsala University, Box 549, 751 24 Uppsala, Sweden

**Keywords:** BmNPV, Probiotics, *Lactobacillus acidophilus*, Thai silkworm, *Bombyx mori*

## Abstract

**Objective:**

*Bombyx mori* nucleopolyhedrovirus (BmNPV) causes grasserie with severe effects in Thai strains of the silkworm *Bombyx mori*. The aim of this study was to investigate the effect of probiotic supplementation on the survival of silkworm larvae challenged with BmNPV.

**Results:**

Silkworm larvae of the Thai polyvoltine strain Samrong was supplemented with commercial probiotic bacteria, *Lactobacillus acidophilus*, on the second day of the 2nd, 3rd, 4th, and 5th instar. When challenged with BmNPV on the second day of the 4th instar, the survival ratio was 92% for larvae supplemented with *L. acidophilus* as compared to 56% for larvae without *L. acidophilus* supplementation. For the larvae that survived until pupation, we determined the growth characters cocooning ratio, larval weight, and pupation ratio, and the economic characters cocoon shell weight and cocoon weight. Growth characters were significantly lower in larvae infected with BmNPV as compared to infected larvae receiving probiotics.

## Introduction

Sericulture is referred to the products generated from silkworms such as *Bombyx mori*. The silk is not only used for making silk cloth, but it can also be used for other purposes: cosmetics, supplementary food, as food and feed, and medical treatments [1–3].

There are two types of *B. mori*, polyvoltine and bivoltine, where the time from egg to adult is 47–60 days and 42–51 days, respectively [4, 5]. Only larvae feed on mulberry leaves. The cocoon is spun by the fifth instar larva before pupation. After mating and laying eggs, the moths die within 4–7 days. Bivoltine silkworms are reared in Japan and China while polyvoltine silkworms are reared in Thailand and other tropical countries such as India, Cambodia, and Brazil. Polyvoltine cocoons are yellow and small, but rich in sericin, which is the gum coating silk fibers that allows the fibers to stick together [6].

Microorganism pathogens such as bacteria, fungi, microsporidia, and viruses cause serious economic losses in silkworm farming. Among them, *Bombyx mori* Nucleopolyhedrovirus (BmNPV): Baculoviridae: *Alphabaculovirus* [7] causes the most severe disease in silkworm farming in tropical countries, Grasserie [8–12]. Even though grasserie is the most destructive disease in Thailand with about 70% to 100% mortality per season [18], there is a lack of knowledge regarding the BmNPV infection in the field population. Kumpratueang [13] randomly collected silkworm larvae from silkworm farms in 14 provinces in the Northeastern part of Thailand and then investigated BmNPV infection by using a DNA hybridization probe. Fourteen percent of first to third instar larvae were found to be infected while the infection rate for instars four and five was 45%.

BmNPV enters silkworms mainly by ingestion of contaminated mulberry leaves [14], but may also spread by horizontal transmission from other infected larvae. The midgut is the main organ of infection, but also the first barrier of defense against the invasion of pathogenic microbes [15]. The midgut fluid in silkworm larvae is alkaline, which dissolves the viral occlusion bodies (OBs) in the alimentary tract [16, 17]. This leads to the release of occlusion-derived virus (ODV) that enters the midgut epithelial cell by passing through the peritrophic membrane. The ODVs are replicated and produced in the nucleus of silkworm larvae cells. The BmNPV infection is acute, the period of infection to sign of disease is approximately 96 h [18]. BmNPV can also persist inside the silkworm as a latent and covert infection [19, 20], but can cause disease symptoms if activated by high or low temperature or if the environment of the silkworm rearing has been changed [21, 22]. The BmNPV infection is obvious in the 4th and the 5th instar, but it is difficult to observe infected larvae in earlier instars [23].

Nowadays, probiotic supplementation is considered to be an alternative method for prevention and integration management of microorganism diseases in animals, which is safe and without side effects [24]. Several species of lactic acid bacteria (LAB) have been extensively studied [25] and found to be beneficial as probiotics. LAB belonging to the genus *Lactobacillus* are commonly used as probiotic bacteria. They have been isolated from both the exogenous and indigenous microbiota of hosts. Recently, probiotics have become common to use in insects produced for food and feed [26]. In bivoltine silkworms, it has been demonstrated that probiotic strains of *Lactobacillus* can induce the innate immunity [27, 28]. In polyvoltine silkworms, probiotic *L. acidophilus* stimulated growth factors leading to an increase in the silk yield and promotion of the silk harvest [29] and *L. plantarum* helped to increase body weight, cocoon, shell, and pupation ratio [30]. *L. casei* decreased the mortality and increased larval weight, cocooning ratio, pupation ratio together with economic characters when larvae were infected with the microsporidium *Nosema bombycis*, which causes pebrine disease [10, 31].

So far, there are no effective tools for inhibition of BmNPV infection. Here, we explore the use of *L. acidophilus* in the supplementary diet to attempt to decrease the effect of BmNPV infection and find significant positive effects.

## Main text

### Materials and methods

#### Silkworm preparation

Eggs of the Thai silkworm strain Samrong were purchased from sericulturists in Borabue district, Thailand, and maintained in the laboratory. Silkworm eggs were incubated at 26–27 °C for 8 days. After the eggs changed color to be dark or brown, they were disinfected by dipping in 3% formalin for 10 min, then they were placed into 75% ethanol for 1 min, and into 95% ethanol for 1 min, respectively. The eggs were placed back into the incubator for continued incubation at 25 °C until hatching. Newly hatched larvae of Samrong were reared by feeding on mulberry leaves under standard rearing conditions at 25–26 °C with 75–80% relative humidity. The silkworm larvae fed on mulberry leaves until second instar.

#### Preparation of *Bombyx mori* nucleopolyhedrovirus (BmNPV)

The BmNPV used in this study was originally isolated from infected silkworm larvae collected from a rearing house in the Mahasarakham province, Thailand. The virus was propagated in third instar silkworm larvae at Silkworm Diseases and Detection Laboratory, Mahasarakham University. Silkworm larvae were infected by dipping mulberry leaves into suspended BmNPV Occlusion Bodies (OB). Three to five days after BmNPV-OB inoculation, the larvae showed typical symptoms of grasserie disease. They appeared yellowish and puffy in appearance and their hemolymph contained numerous polyhedra of the virus exudated from the wounds. The larvae died within 5–7 days. Dead larvae were collected and homogenized in distilled water, and the homogenate was filtered through 4 layers of cheese cloth. The OBs were pelleted by centrifugation. The concentration of OBs was determined by hemocytometer (counting chamber) and it was diluted to be 10^6^ OBs/ml.

#### Preparation of probiotic bacteria

*Lactobacillus acidophilus* strain TISTR 1443 was purchased from Thailand Institute of Scientific and Technological Research (TISTR). It was streaked out at Luria Bertani agar (LB) agar (2% NaCl) and incubated overnight at 37 °C. A single colony was picked up and transferred into 10 ml of LB broth, supplemented with 2% NaCl (w/v). The culture was then incubated at 28 °C overnight with agitation. The *L. acidophilus* culture was transferred again into 50 ml of Man, Rogosa, Sharpe (MRS) medium broth and incubated overnight at 28 °C. The culture broth was then centrifuged at 5,000 rpm and 4 °C for 15 min. The pellet of *L. acidophilus* was collected and washed with distilled water. The suspended culture used to feed the silkworms (10^8^ bacteria/ml) was counted by the counting chamber method [32].

#### Feeding experiment

To investigate the effects of probiotics on BmNPV-OB infection, silkworm larvae were fed on mulberry leaves cut in pieces of 2 × 2 cm and dipped in *L. acidophilus* (10^8^ bacteria/ml), followed by mulberry leaves dipped in BmNPV-OB (10^6^ PIBs/ml). Four treatments were set up: (1) larvae fed on mulberry leaves only, (2) larvae fed on mulberry leaves supplemented with *L. acidophilus* (10^8^ bacteria/ml), (3) larvae fed on mulberry leaves supplemented with *L. acidophilus* (10^8^ bacteria/ml) and BmNPV-OB, (4) larvae fed on mulberry leaves supplemented with BmNPV-OB. Mulberry leaves supplemented with *L. acidophilus* were given on the second day after molting in the 2nd, 3rd, 4th and the 5th instar (thus at day 5, 8, 12 and 16). Mulberry leaves supplemented with BmNPV—OB were given on the second day after molting in the 4th instar. Each experiment was replicated three times with twenty-five silkworm larvae per replication. The number of deceased larvae were counted daily until the end of the 5th instar. The survival ratio (%) of larvae were counted and calculated. The quality parameters such as growth characters; larval weight of the 5th instar (g), cocooning ratio (%), pupation ratio (%), and economic characters; cocooning weight (g) and cocoon shell weight (g) were recorded and determined.

#### Statistical analysis

The data obtained from the experiments were analyzed using the Duncan multiple range test at 95% confidence level with the Statistica 4.3 program.

## Results

### Confirmation of grasserie infection

**Fig. 1:**
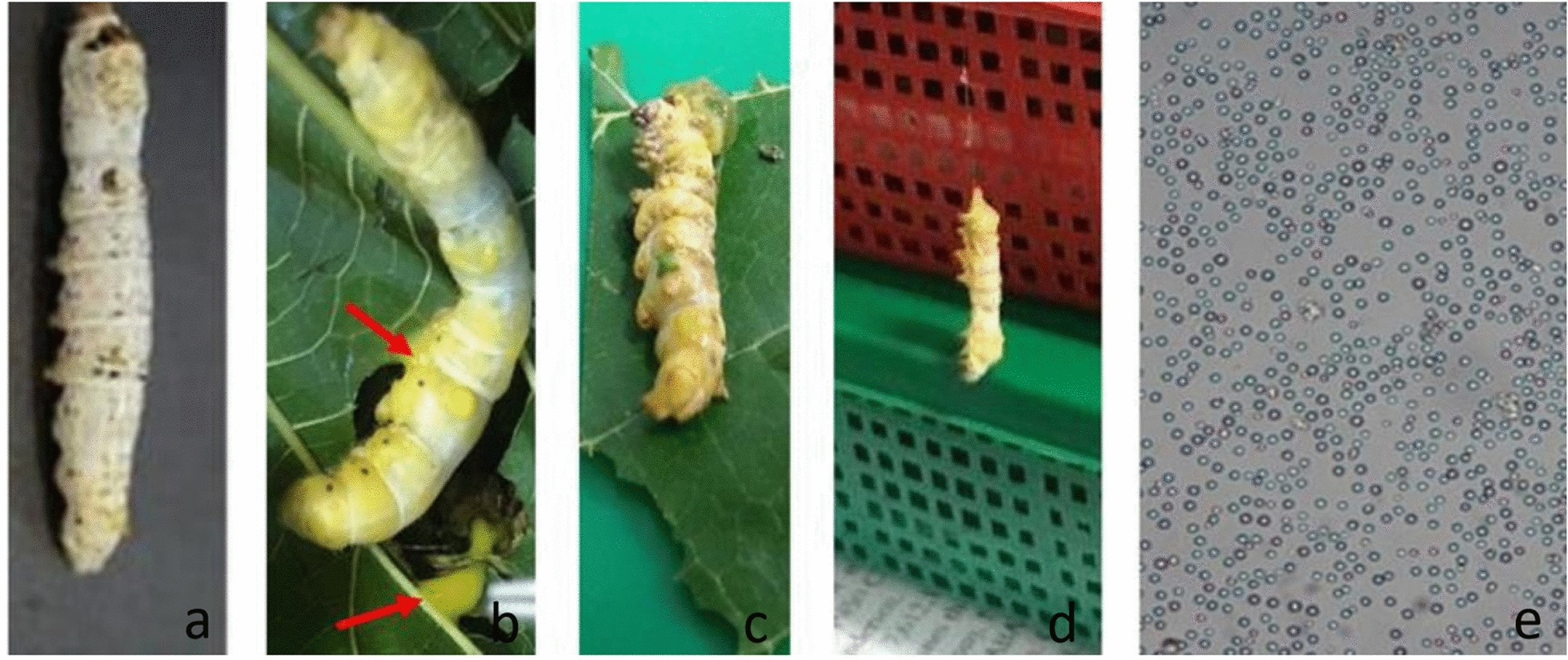
The symptoms of grasserie disease caused by *Bombyx mori* nucleopolyhedrovirus in the 5th instar larva, **a** healthy larva, **b** severely infected larva appearing yellowish in body color with turbid hemolymph containing BmNPV– OB (arrows), **c**, **d** cadavers of diseased larvae whereof **d** is hanging showing so-called tree top disease, and **e** Occlusion Bodies (OBs) observed under light microscope (40X magnification)

Confirmation of grasserie infection in the Thai silkworm Samrong was based on the symptom of grasserie and the presence of BmNPV- OBs (Fig. 1). The infected larvae showed grasserie symptoms after 5–7 days after ingestion of OBs. The larvae occurred yellowish and soften, the hemolymph exuded from the wounds (Fig. 1b–d.). To confirm the presence of OBs, the silkworm prolegs were cut and the hemolymph was collected and analyzed by light microscopy. The OBs appeared as small spots with circular shape (Fig. 1e).

### Effects of probiotics and BmNPV on survival ratio of *B. mori*

**Fig. 2:**
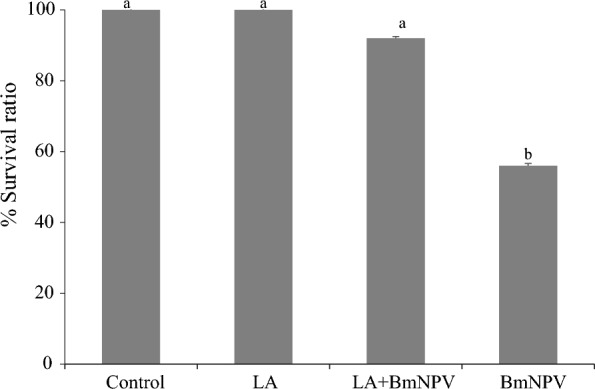
The effects of *L. acidophilus* (LA) and BmNPV on survival ratio of *B. mori* larvae. Treatments were performed with and without 10^8^ cells/mL of *L. acidophilus* and 10^6^ OBs/mL of BmNPV and the results were compared to those of the control group, which were fed with distilled water. Different letters on the top of each bar represent difference identified by Analysis of Variance coupled with Duncan’s Multiple Range Test at 95% significant level. Bars represent 1 unit of standard deviation

Treatments were performed with and without 10^8^ cell/ml of *L. acidophilus* and 10^6^ OBs/ml of BmNPV and the results were compared to those of the control group, which were fed with distilled water. The feeding experiments resulted in a statistically significant difference in survival ratio for BmNPV-infected larvae supplemented with probiotics (92.00 ± 0.33%) as compared to BmNPV infected larvae not receiving probiotics (56.00 ± 1.45% Fig. 2).

There was no difference in survival ratio for silkworm larvae supplemented with *L. acidophilus* (100.00 ± 0.33%), control larvae, and larvae receiving both *L. acidophilus* and BmNPV (92.00 ± 0.33%).

### Effects of probiotics and BmNPV on quality parameters

The quality parameters constituted of growth characters: larval weight, cocooning ratio and pupation ratio, and economic characters: fresh cocoon weight (cocoon + pupa), and cocoon shell weight (cocoon without pupa). The larvae receiving *L. acidophilus* had significantly higher larval weight, pupation ratio, cocooning ratio, and cocoon shell weight as compared to the control group (Table 1).

**Table 1:** Effects of *Lactobacillus acidophilus* on BmNPV-challenged Thai polyvoltine strain, Samrong

Treatments	Quality parameter				
	Growth characters			Economic characters	
	Larval weight of 5th instar (g)	Cocooning ratio (%)	Pupation ratio (%)	Cocoon weight (g)	Cocoon shell weight (g)
BmNPV-OB (10^6^ OB/ml)	2.12 ± 0.96^a^	92.86 ± 2.00^a^	76.92 ± 1.53^a^	1.07 ± 0.33^a^	12.15 ± 3.63^a^
*L. acidophilus* (10^8^ cell/ml) followed by BmNPV-OB (10^6^ OB/ml)	2.80 ± 0.96^c^	95.83 ± 0.33^b^	95.65 ± 0.33^b^	1.40 ± 0.00^c^	16.43 ± 4.76^a^
*L. acidophilus* (10^8^cells/ml)	2.62 ± 0.48^bc^	96.00 ± 0.00^b^	100 ± 0.33^b^	1.32 ± 0.33^b^	12.88 ± 2.27^a^
No treatment	2.26 ± 0.64^ab^	92.00 ± 0.33^b^	95.65 ± 0.33^b^	1.23 ± 0.44^b^	16.26 ± 1.79^a^

Quality parameters of pupae from surviving larvae. Statistically significant differences as measured by Duncan multiple range test are denoted with different letters

As compared to larvae receiving only BmNPV, the 5th larval instar weight was significantly higher in all other groups. BmNPV infection significantly lowered the cocooning ratio, but probiotic treatment increased the ratio to an intermediate level. The pupation ratio was 100% in the groups not infected by BmNPV. The probiotic treatment resulted in a significantly higher pupation ratio in BmNPV-infected larvae (95% with probiotics vs. 76% without probiotics). The highest cocoon weight and cocoon shell weight were obtained in those larvae receiving probiotics, although this difference was not statistically significant.

## Discussion

The Thai silkworm strain Samrong is provided to Thai farmers because of its high silk yield. It has a short life cycle, which results in a short time to obtain silk products. Moreover, being polyvoltine it can be reared all year around [33]. However, a major problem for the silk production in Thailand is diseases of silkworm larvae. Grasserie disease caused by BmNPV (*Alphabaculovirus*: Baculoviridae) is the most destructive disease in silkworm rearing in tropical regions such as Thailand. The BmNPV infection leads to an acute disease and also exists as latent and covert infections [19, 20].

Probiotics used as supplementation have been shown to inhibit and reduce growth of pathogens, for example in honey bees [34]. Also in silkworms it has been shown that the innate immunity was stimulated by the probiotic lactic acid bacteria, in this case *Lactococcus lactis* [27]. Several studies on the effect of probiotics on virus defense have been conducted in shrimp where the white spot syndrome virus (WSSV) is a key pathogen ([35] and references therein). The innate immune system is similar in different groups of arthropods, and therefore one can expect similarities regarding the effect of probiotics in *Bombyx mori* as compared to shrimps. In general, the arthropod responds to the probiotics with an enhanced immune response that in part is affecting the virus. Arthropods react to the invasion of bacteria with the help of pattern recognition molecules, but since the probiotic bacteria are harmless to the host, the activated innate immune system provides the host with virus protection. Among the responses to the invasion are transcription of immune-related genes, production of phenoloxidase leading to melanization, activation of reactive oxygen (NOs), and stimulated production of hemocytes. The hemocytes are responsible for encapsulation, nodule formation and phagocytosis. In this paper, we treated *Bombyx mori* larvae with probiotics as an attempt to interfere with the BmNPV infection. The treatment was a success and BmNPV-exposed silkworm larvae survived at the same level as uninfected larvae when supplemented with *L. acidophilus* at a concentration of 10^8^ cells/ml. At this stage, it remains to investigate which of these immune responses that are active in rescuing the silkworm larvae.

For resistance/tolerance or susceptibility to pathogens the midgut environment of the silkworm larva plays a major role. Degradation of BmNPV—Occlusion bodies is most effective at pH 9–10 [36, 37]. Thus, the decrease in number of dead silkworm larvae after treatment with lactic acid bacteria could be related to the creation of a lower pH in the midgut. Alternatively, a competitive adhesion to the intestinal mucosa, which would inhibit pathogen adhesion, and modulation of the immune system (see above) could affect the BmNPV infection [38].

In our study, BmNPV-infected larvae without probiotic treatment had a significant weight loss as compared to all other groups. BmNPV-infected larvae receiving *L. acidophilus* had a significant weight gain as compared to the infected larvae, but also to the non-infected larvae. Thus, the probiotic treatment seemed to increase food digestion and/or absorption of nutrients, which promoted growth characters. Similarly, it has been shown that *L. casei* positively affect growth characters in *B. mori* [10, 39]. One reason for increased weight gain in silkworm larvae, apart from the effect of probiotics on the silkworm health status, is the ability for *L. casei* to help breaking down mulberry leaves more effectively [40], allowing the silkworms to extract and utilize nutrients efficiently, leading to faster growth and increased body weight.

This is the first report of using probiotics to reduce the mortality of *B. mori* challenged by BmNPV. Understanding the mechanism of how probiotic treatment affects the BmNPV infection should be a future priority.

## Limitations

We have in this study showed that the mortality of silkworm larvae infected by BmNPV can be reduced by supplementation of *Lactobacillus acidophilus*. To elucidate the role and mechanism of this effect would require more investigations. For example, is the protective effect caused by a lower pH, and if so, for how long does it remain after supplementation of bacteria? Is the potential release of virus particles from the occlusion bodies hindered directly or indirectly by (the presence of) *L. acidophilus*? How much bacteria are needed and when should they be applied for optimal results?

This study has been conducted with one strain of bacteria, virus and host. It has also focused on only one concentration of BmNPV and bacteria. While this proof-of-principle is a promising first step towards an efficient proactive treatment of silkworms, much work is required for developing an optimal use of *Lactobacillus* in silkworm rearing.

## Data Availability

The datasets used and/or analyzed during the current study are available from the corresponding author on reasonable request.

## Abbreviations

BmNPV: *Bombyx mori* Nucleopolyhedrovirus
OB: Occlusion Body
LB: Luria Broth
MSU: Mahasarakham University
TISTR: Thailand Institute of Scientific and Technological Research
